# Identification of *NPF* Family Genes in *Brassica rapa* Reveal Their Potential Functions in Pollen Development and Response to Low Nitrate Stress

**DOI:** 10.3390/ijms24010754

**Published:** 2023-01-01

**Authors:** Xiaoshuang Yang, Wenyu Han, Jiao Qi, Yueying Li, Xingbo Chen, Yiwen Zhang, Jingyu Wu, Genze Li, Jing Gao, Xiangshu Dong

**Affiliations:** 1 School of Agriculture, Yunnan University, Kunming 650091, China; 2 Industrial Crop Research Institute, Yunnan Academy of Agricultural Sciences, Kunming 650205, China

**Keywords:** *Brassica rapa*, NPF gene family, low nitrate stress, pollen development

## Abstract

*Nitrate Transporter 1/Peptide Transporter Family* (*NPF*) genes encode membrane transporters involved in the transport of diverse substrates. However, little is known about the diversity and functions of NPFs in *Brassica rapa*. In this study, 85 *NPFs* were identified in *B. rapa* (*BrNPFs*) which comprised eight subfamilies. Gene structure and conserved motif analysis suggested that *BrNFPs* were conserved throughout the genus. Stress and hormone-responsive *cis*-acting elements and transcription factor binding sites were identified in *BrNPF* promoters. Syntenic analysis suggested that tandem duplication contributed to the expansion of *BrNPFs* in *B. rapa*. Transcriptomic profiling analysis indicated that *BrNPF2.6*, *BrNPF2.15*, *BrNPF7.6*, and *BrNPF8.9* were expressed in fertile floral buds, suggesting important roles in pollen development. Thirty-nine *BrNPFs* were responsive to low nitrate availability in shoots or roots. *BrNPF2.10*, *BrNPF2.19*, *BrNPF2.3*, *BrNPF5.12*, *BrNPF5.16*, *BrNPF5.8*, and *BrNPF6.3* were only up-regulated in roots under low nitrate conditions, indicating that they play positive roles in nitrate absorption. Furthermore, many genes were identified in contrasting genotypes that responded to vernalization and clubroot disease. Our results increase understanding of *BrNPFs* as candidate genes for genetic improvement studies of *B. rapa* to promote low nitrate availability tolerance and for generating sterile male lines based on gene editing methods.

## 1. Introduction

Nitrate Transporter 1/Peptide Transporter (NRT1/PTR) family proteins are transporters of the Major Facilitator Superfamily (MFS) and are referred to as NPF (NRT1/PTR family) proteins that are present in all major domains of life [1]. The first identified *NPF* gene in plants was *AtNPF6.3*/*AtNRT1.1*/*CHL1* (*CHLORINA 1*), and the protein was functionally characterized as a nitrate transporter [2]. Subsequently, nitrate has been considered the primary substrate of *NPFs*. However, further identification of *NPFs* from various plants has demonstrated that NPFs can transport a wider range of substrates, including nitrate, chloride, oligopeptide, IAA (Auxin), JA (Jasmonate), GA (Gibberllin), abscisic acid (ABA), glucosinolates, potassium, and sugar [3,4,5,6,7,8,9]. Further, some NPFs can transport multiple substrates, such as *AtNPF6.3*/*AtNRT1.1*, that has reported dual-affinity nitrate transport activity (i.e., response to both low- and high-nitrate concentrations) and is also involved in auxin transport [3,8].

During plant development and responses to environmental stresses, nutrients and other substrates are transported according to altered metabolic pathways involved in the synthesis, storage, mobilization, and conversion, thereby requiring transporters with capacities to transport a wide diversity of chemical substrates [10,11]. NPFs are one of the largest transporter groups in plants and can transport a wide range of substrates. For example, AtNPF2.10/GTR1 (glucosinolate transporters 1) and AtNPF2.11/GTR2 are essential for the translocation of glucosinolate defense compounds to seeds during maturation [5]. Furthermore, AtNPF2.3 functions as a root stele transporter that contributes to nitrate translocation to shoots during salt stress [12]. In addition, AtNPF2.8/FST1 (Flavonol-Sophoroside-Transporter 1) is expressed in the tapetum and is required for the accumulation of flavonol glycosides on pollen surfaces [13]. ZmNPF7.9 in maize promotes lipid and amino acid homeostasis activity during seed development, while ZmSUGCAR1 (*Zea mays* Sucrose and Glucose Carrier 1) is a paralog of AtNPF7.3 and is expressed in the basal endosperm transfer layer (BETL) of maize kernels, where it acts as a sugar transporter that imports glucose and sucrose into the endosperm [9,14]. Whenaken together, these observations suggest that NPFs play key roles in plant development and are responsive to environmental stresses, owing to their broad substrate specificity.

Given the important roles of NPFs in plants and the increasing availability of plant genomes, the systematic identification, and analysis of *NPF* genes have been conducted in many plants, leading to the identification of 53 *NPFs* in *Arabidopsis* [15], 80 in rice [16], 73 in apple [17], 178 in sugarcane [18], 169–199 in *Brassica napus* [19,20,21], 331 in hexaploid wheat (*Triticum aestivum* L.) [22], 57 in Spinach (*Spinacia oleracea* L.) [23], and 109 in Tea Plants (*Camellia sinensis*) [24]. Phylogenetic analysis has consistently divided *NPFs* into eight subfamilies, termed *NPF1* to *NPF8* [1]. Using *Arabidopsis* as a model, AtNPF1 and AtNPF2 from the NPF1 subfamily (comprising three members) are involved in the redistribution of nitrate into developing leaves [25]. The NPF2 family comprises 14 members that can transport numerous substrates, including nitrate, glucosinolates, and phytochromes [26]. Only one *NPF* gene, *AtNPF3.1*, is in the *Arabidopsis* NPF3 subfamily and functions in nitrite and GA transport [27,28]. Seven members comprise the NPF4 subfamily, and some can transport ABA and GA in addition to other substrates [4,28]. The NFP5 subfamily comprises 16 members that participate in the transport of nitrate, dipeptides, GA, JA, and ABA [29,30]. Four members comprise the NPF6 subfamily, including the first characterized *NPF* gene, *AtNPF6.3*, which primarily transports nitrate [31,32]. Three members comprise the NPF7 subfamily, all of which can transport nitrate [33,34,35], although cadmium and sodium could also be substrates of *AtNPF7.2* and *AtNPF7.3* [33,34]. Lastly, the NPF8 subfamily comprises five members, with some of them able to transport JA and dipeptides [36,37]. Notably, half of the AtNPFs with characterized functions are capable of transporting nitrate [35,38].

*Brassica rapa* is one of the most important crops in China, Korea, and other Asian countries, including several oil seed crops such as sarson and turnip rape, in addition to numerous vegetable crops such as turnip, Pak-choi, and Chinese cabbage [39]. The *NPFs* of *B. rapa* were previously isolated to investigate the functions of *BnaNPFs* in *B. napus* [19,20,21], although detailed characterizations of *BrNPFs* remain limited. In this study, *BrNPFs* were systematically identified and characterized from three *Brassica* genomes, with analysis of their gene composition, chromosomal locations, phylogenetic relationships, and the presence of *cis*-elements in their promoters. In silico and Semi-quantitative RT-PCR analyses indicated that *BrNPF2.6*, *BrNPF2.15*, *BrNPF7.6*, and *BrNPF8.9* could be related to pollen development, as reflected by the co-expression of genes related to these functions. The responses of *BrNPFs* due to vernalization and clubroot were also investigated, while the expression of *BrNPFs* under low nitrate stress was evaluated using transcriptomic data. The results from this study provide a framework for better understanding the functions of *BrNPFs* during pollen development and *B. rapa* responses to vernalization, clubroot disease, and low nitrate stress.

## 2. Results

### 2.1. Bioinformatics Analysis of NPFs

#### 2.1.1. Identification of NPF Proteins in Three Prototypical Diploid Species of Brassica

A total of 85, 110, and 97 NFP proteins were identified in the genomes of *B. rapa*, *B. oleracea*, and *B. nigra*, respectively (Table S1). The numbers of Br/Bol/BniNPF proteins were 1.6, 2.0, and 1.8 times that in *Arabidopsis*, respectively, owing to the gene expansion of *Brassica* during their evolution. The gene IDs, genome locations, coding sequence lengths, protein lengths, and other characteristics of the identified *Br/Bol/BniNPF* genes are listed in Table S1. The subcellular localization of these 292 proteins were predicted using the WoLF PSORT program, revealing that most of the NPF proteins were located on the plasma membrane (269), chloroplast (11), or in vacuoles (8) (Table S1). The lengths of BrNPFs ranged from 98 to 1070 amino acids, with an average length of 534 amino acids, and MWs ranging from 8.77 to 383.66 kDa, with an average weight of 62.08 kDa. Likewise, a wide range of PIs was also observed (4.73–9.74) which may be due to the considerable range in protein lengths (Table S1).

#### 2.1.2. Phylogenetic, Conserved Motif, and Gene Structure Analysis of BrNFPs

**Figure 1:**
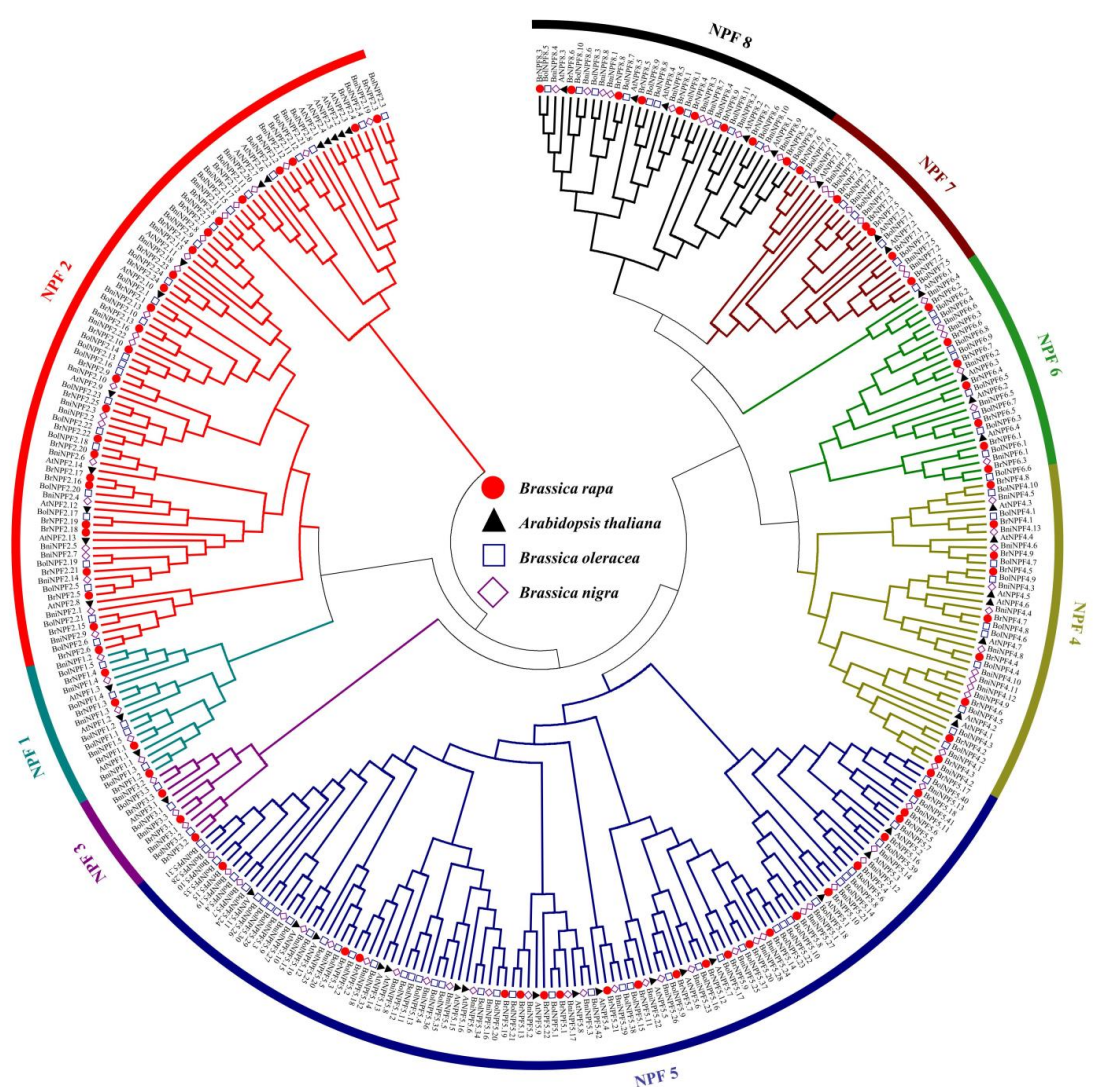
Phylogenetic reconstruction of 345 NPF proteins identified in the genomes of *Brassica rapa* (n = 85), *B. oleracea* (110), *B. nigra* (97), and *Arabidopsis* (53). The neighbor-joining phylogenetic tree was generated in MEGA6 with full-length NPF protein sequences, and branch support was evaluated with 1000 bootstrap replicates.

In order to explore the evolutionary characteristics and classification of BrNPF proteins, phylogenetic analysis with neighbor-joining methods was conducted with 292 Br/Bol/BniNPF protein sequences and 53 AtNPFs. All NFP proteins were classified into eight subfamilies, based on the scheme presented in [1], and belonged to the families NPF1-8 (Figure 1). The NPF5 subfamily comprised the most *NFP* genes (109 members), followed by the subfamilies NPF2 (85 members), NPF4 (39 members), NPF8 (35 members), NPF6 (27 members), NPF7 (23 members), NPF1 (17 members), and NPF3 (10 members). An ortholog in the *Brassica* genomes was not found in the subfamily NFP2 for AtNPF2.1, AtNPF2.2, and AtNPF2.5, indicating gene loss during evolution (Figure 1). Six homologs of AtNPF5.2 were identified in *B. rapa* (BrNPF5.4, BrNPF5.5, BrNPF5.6, BrNPF5.16, BrNPF5.17, and BrNPF5.18), six in *B. oleracea* (BolNPF5.6, BolNPF5.7, BolNPF5.8, BolNPF5.39, BolNPF5.40, and BolNPF5.41) and four in *B. nigra* (*BniNPF5.11*, *BniNPF5.12*, *BniNPF5.13*, and *BniNPF5.14*) (Figure 1), indicating the expansion of AtNPF5.2 in three prototypical *Brassica* diploid species over evolutionary time.

**Figure 2:**
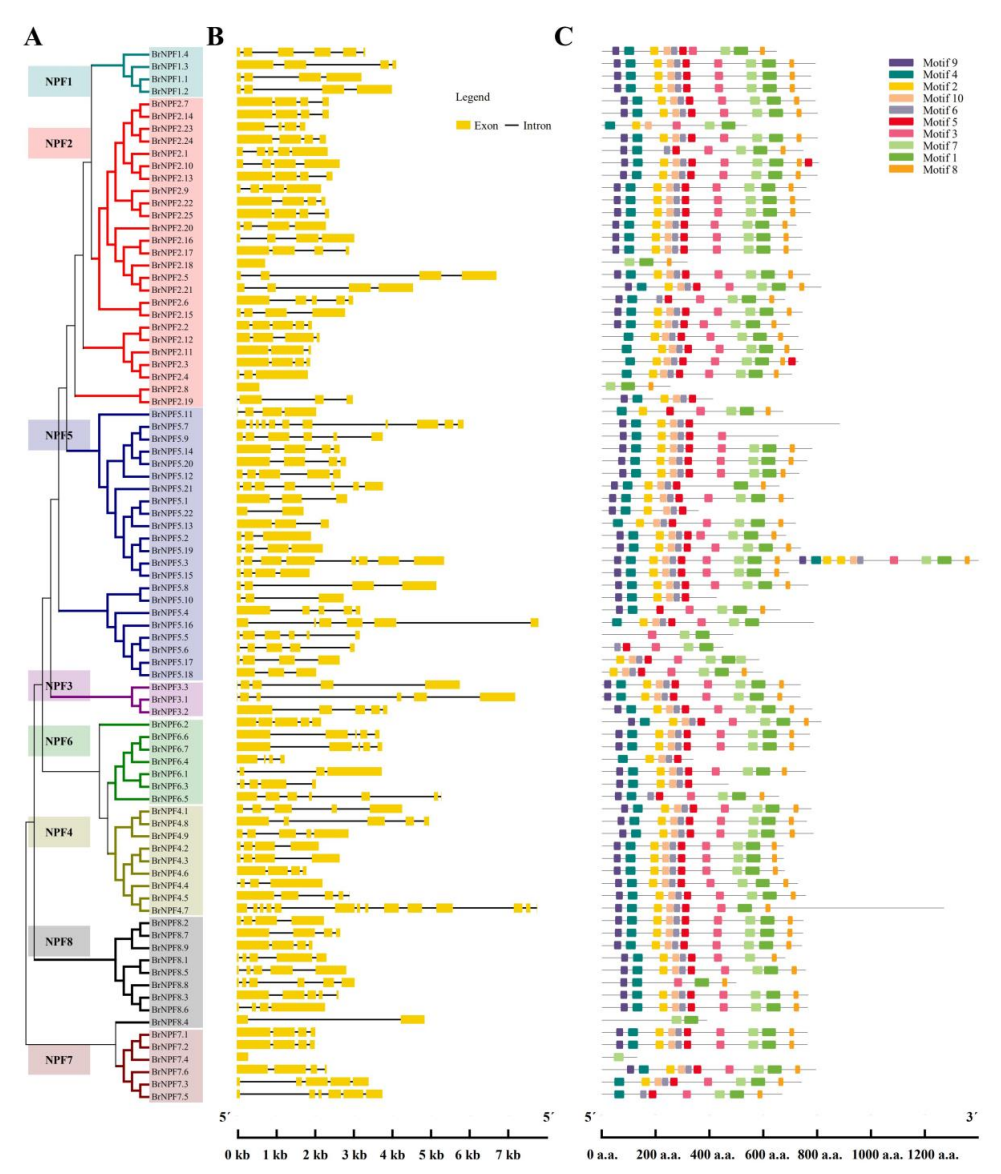
Phylogenetic tree (**A**), gene structures (**B**), and MEME motifs (**C**) for 85 NPFs identified in *B. rapa*. (**B**) Yellow boxes represent exons, and black lines indicate introns. (**C**) The colored boxes indicate motifs, as shown by the legend on the right.

The conserved motifs of the BrNPFs were analyzed with the MEME program, yielding the identification of 10 conserved motifs among 85 BrNPFs from *B. rapa* (Figure 2 and Table S2). The 10 motifs of typical BrNPF proteins followed the order of Motif9-Motif4-Motif2-Motif10-Motif6-Motif5-Motif3-Motif7-Motif1-Motif8. Apart from motifs 7, 8, and 9, the other motifs contained the core PTR2 domain conserved sequence (Table S3). No significantly conserved motifs were identified for the 7, 8, and 9 domains based on BLAST searches with NCBI and Pfam.

In order to investigate the structural diversity of *BrNPF*s, exon-intron organizations were analyzed. The number of BrNPF introns ranged from 0 to 14. The most common exon-intron organization comprised four exons separated by three introns, which were present in 41 *BrNPFs* (Figure 2 and Table S2). Except for *BrNPF2.8*, *BrNPF2.18*, and *BrNPF7.4*, most *BrNPFs* contained more than one intron, indicating the possible existence of alternative splicing during expression. Eight types of gene structures were identified in the NPF5 subfamily, implying they exhibited diverse functions. *BrNPF* members from the same subgroups exhibited similar gene structures for the other subfamilies, indicating the potential for conserved functions (Figure 2).

#### 2.1.3. BrNPF Chromosomal Location and Gene Duplication Analysis

**Figure 3:**
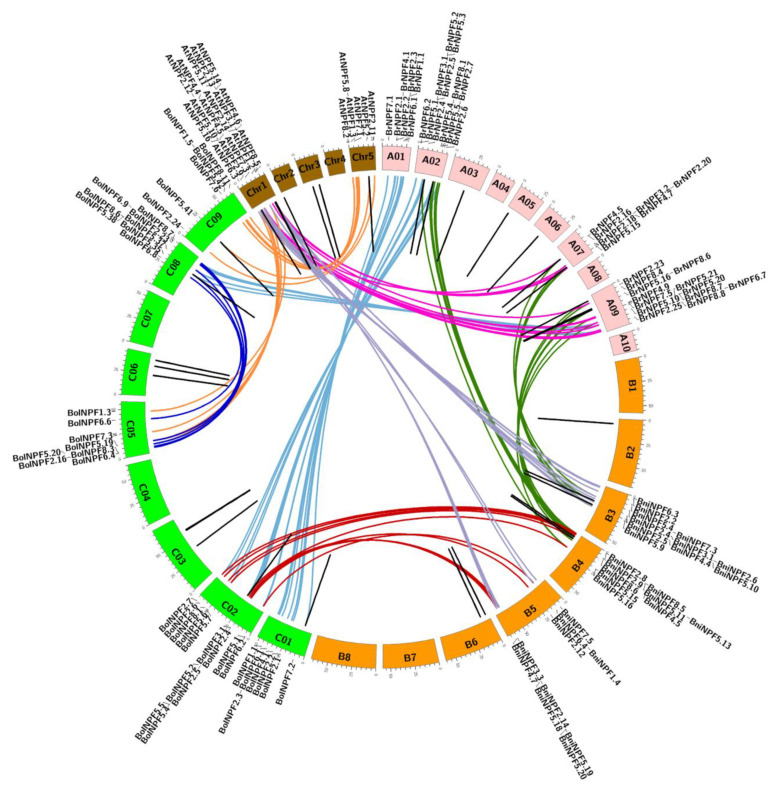
Syntenic relationships among NPF genes of *Brassica rapa*, *B. nigra*, *B. oleracea*, and *Arabidopsis*. The chromosomes of *B. rapa* (chromosomes A01 to A10), *B. nigra* (B1 to B8), *B. oleracea* (C01 to C09), and *Arabidopsis* (Chr1 to Chr5) are shown in red, orange, green, and brown, respectively. Collinear gene pairs were mapped onto chromosomes and are connected to each other. The collinear gene pairs from *B. rapa* and *B. oleracea*, *B. rapa* and *B. nigra*, *B. rapa* and *Arabidopsis*, *B. nigra* and *B. oleracea*, *B. nigra* and *Arabidopsis*, *B. oleracea* and *Arabidopsis* are connected by light blue, green, pink, red, purple, and orange lines, respectively. Segmental duplication genes in *B. oleracea* are connected by dark blue lines. The locations of tandem duplicated genes are indicated with black lines. Only the names of collinear gene pairs are shown.

**Figure A1:**
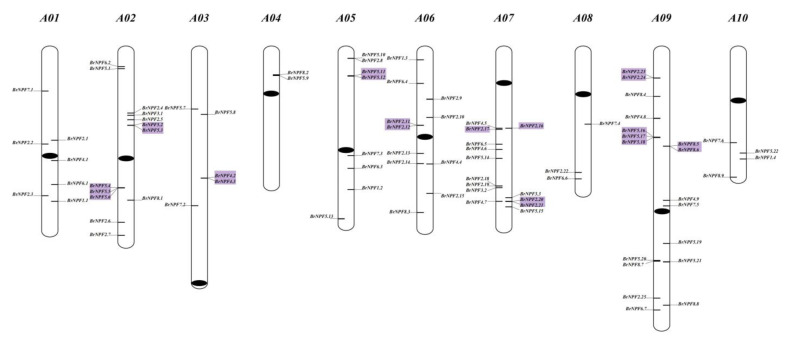
Chromosomal locations of *BrNPFs* identified in this study. The chromosomal number is indicated above each chromosome. Black ovals on each chromosome indicate centromeric regions. Tandem duplicated genes are indicated by purple rectangles.

Eighty-five *BrNPF* genes were present on the ten chromosomes of *Brassica rapa* and were non-uniformly distributed (Figure A1). Chromosome A09 harbored the largest number of *BrNPFs* (18 members), followed by chromosomes A07, A02, and A06, which contained 14, 13, and 11 *BrNPFs,* respectively. Chromosome A04 carried the smallest number of *BrNPFs* (two). BLAST and MCScanX analysis indicated that *BrNPF* gene duplication events were present in the *B. rapa* genome. Briefly, 22 tandem duplicated genes (25.9%) were identified that belonged to ten clusters (Figure 3 and Figure A1). Among the tandem duplicated genes, two, two, and three clusters were located on chromosomes A02, A07, and A09, respectively. The other three clusters were located on chromosomes A03, A05, and A06, respectively (Figure 3 and Figure A1). These results suggest that tandem duplication is related to *NPF* expansion in *Brassica* genomes.

To evaluate the collinear relationships of all *NFP* genes in *Arabidopsis*, *B. rapa*, *B. nigra*, and *B. oleracea*, collinear gene pairs were identified using the MCScanX software package. A total of 26, 18, and 13 gene pairs were identified between *B. rapa* and *B. oleracea*, *B. rapa* and *B. nigra*, and *B. rapa* and *Arabidopsis*, respectively (Figure 3 and Table S3). All syntenic *NPF* genes in *B. rapa* were located on chromosomes A01, A02, A07, and A09 (Figure 3). Six segmental duplication events were also identified within the *B. oleracea* genome (Figure 3), indicating that the greater numbers of *NPFs* in the *B. oleracea* genome could be due to segmental gene duplication events in its genome.

#### 2.1.4. Cis-Elements in Promoters of BrNPFs

In order to identify the *cis*-regulatory elements in *BrNPF* promoters, cis-elements were analyzed using the PlantCARE platform (https://bioinformatics.psb.ugent.be/webtools/plantcare/html/ accessed on 16 August 2022). A total of 904 phytohormone-responsive elements, 1605 environmental-responsive elements, 205 plant growth, and development-related elements, and 979 transcriptional factor binding sites were predicted within the promoters of the 85 *BrNPFs* (Table S4). Among these, light-responsive, MYB transcription-binding, MYC transcription-binding, and ABA-responsive *cis* elements were the four most prevalent (Table S4). Thus, most *BrNPFs* respond to diverse environmental stresses and are regulated by various transcriptional factors (TFs).

### 2.2. Tissue Expression of BrNPFs Reveals Their Potential Functions during Pollen Development

**Figure 4:**
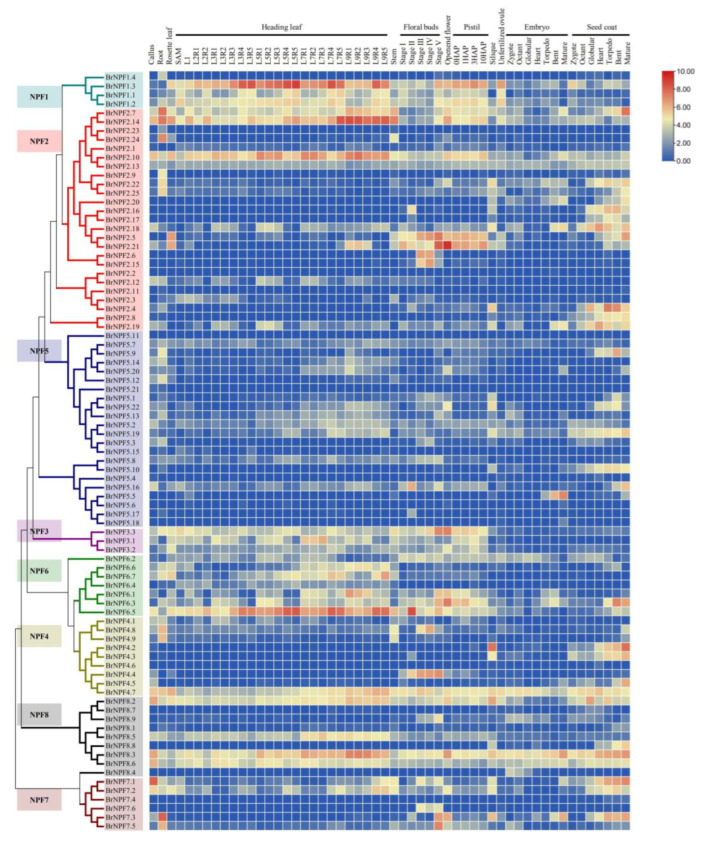
Expression of *BrNPFs* in different tissues and organs of *Brassica rapa*. Expression data were subjected to log_2_(TPM+1) normalization. Comparisons of stem leaf, stem, and root tissues were conducted by comparison against seven-week-old Chiifu (*B. rapa* cv. Chiifu) plants. Flower tissue was also generated from blooming plants without floral shoots, while silique tissues were obtained 15 days after pollination. Further, 24 samples from the heading leaves of eleven-week-old plants were collected. All leaves from the heading leaf samples were divided into eleven whorls extending from the inside to the outside. Leaves with a length < 2 cm and shoot apical meristem (SAM) are identified as SAMs. Leaves from whorls one, two, three, five, seven, and nine were identified as L1, L2, L3, L5, L7, and L9, respectively. L2 samples were divided into leaf petiole (L2R2) and leaf blade (L2R1) samples, while L3, L5, L7, and L9 were divided into regions including the top region (R1), outer margin region (R2), the middle region of the blade (R3), top region of the petiole (R4), and middle region of the petiole (R5). FS1-5 and SS1-5 indicate the floral buds from the ‘Bcajh97-01A/B’ GMS A/B line of *B. rapa*, representing the pollen mother cells, tetrad, uninucleate pollen, binucleate pollen, and mature pollen stages, respectively. 1 HAP, 3 HAP, and 10 HAP indicate pistils at 1, 3, and 10 h after pollination in the fertile line, respectively.

**Figure 5:**
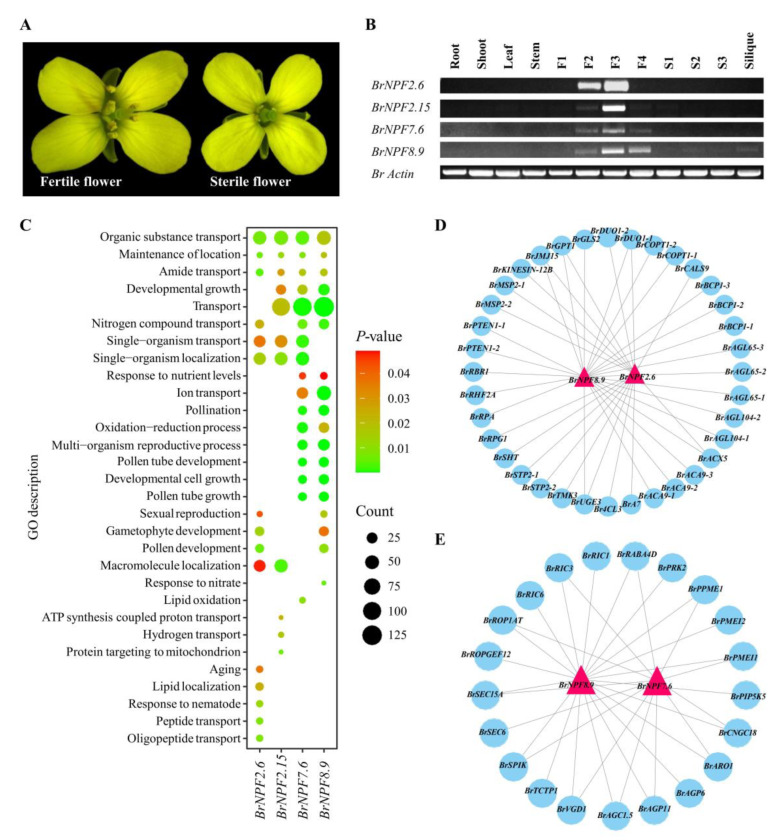
Confirmation and analysis of *BrNPFs* related to pollen development. (**A**), The phenotype of male *Brassica rapa* sterile lines. (**B**), Semi-quantitative RT-PCR analysis of *BrNPFs* related to pollen development within various tissues. (**C**), GO enrichment analysis of co-expression of *BrNPFs*. (**D**), Network of pollen development-related genes based on co-expression analysis of *BrNPF8.9* and *BrNPF2.6*. (**E**), Network of pollen tube development-related genes based on co-expression analysis of *BrNPF8.9* and *BrNPF7.6*.

In order to investigate the expression of *BrNPFs*, their expression patterns were compared using RNA-sequencing data from 59 different organ or tissues samples, including callus, roots, stems, stem leaves, flowers, siliques, head leaves (24 samples), developmental stages of floral buds (10 samples), pistils (four samples), unfertilized ovules, embryos (seven samples), and seed coats (seven samples) (Table S5). *BrNPFs* were differentially expressed among groups of the 59 tissue samples (Figure 4). The *BrNPF1.1*, *BrNPF1.2*, *BrNPF1.3*, and *BrNPF6.5* genes were generally predominantly expressed in all tissues except embryos and seed coats. *BrNPF2.5* and *BrNPF2.21* were mostly expressed in stem leaves and opened flowers. *BrNPF7.3* and *BrNPF7.5* were predominantly expressed in roots, while *BrNFP7.1* was predominantly expressed in callus tissues. *BrNPF2.5* and *BrNPF2.21* were mostly expressed in all developmental floral buds and late development seed coats. Further, *BrNPF2.6*, *BrNPF2.15*, *BrNPF7.6*, and *BrNPF8.9* were predominantly expressed in fertile buds from the uninucleate to binucleate pollen stages. The primary difference between fertile and sterile floral buds is the presence of pollen grains [40]. Accordingly, *BrNPF2.6*, *BrNPF2.15*, *BrNPF7.6*, and *BrNPF8.9* were predominantly expressed per normal pollen development in *B. rapa*. Semi-quantitative RT-PCR was conducted to confirm the expression patterns of these four genes based on the different developmental stages of floral buds from male genetic sterility (GMS) lines (Figure 5A,B). The expression patterns of these four genes were similar between RNA-Seq and RT-PCR datasets. Briefly, *BrNPF2.6*, *BrNFP7.6*, and *BrNPF8.9* were specifically expressed in the floral buds from F2 (floral buds containing tread stage pollen) to F3 (floral buds after the tetrad stage, but before containing mature pollen) stages (Figure 5B). In addition, *BrNPF2.15* was only specifically expressed in F3 floral buds (Figure 5B).

In order to identify the functions of *BrNPF2.6*, *BrNPF2.15*, *BrNPF7.6*, and *BrNPF8.9* during pollen development, co-expression analysis was conducted with the RNA-seq data from five different types of sterile male lines [41,42,43,44]. Using a Pearson correlation coefficient (PCC) value criterion between -0.6 and 0.6, a total of 1627, 1796, 1829, and 2309 genes were co-expressed with *BrNPF2.6*, *BrNPF2.15*, *BrNPF7.6*, and *BrNPF8.9*, respectively (Table S6). GO enrichment analysis was subsequently conducted to assess biological processes related to these co-expressed genes (Figure 5C). Pollen development, gametophyte development, and sexual reproduction were represented by the genes co-expressed with *BrNPF2.6* and *BrNPF8.9* (Figure 5C). In addition, pollen tube development, pollination, and pollen tube growth processes were represented by genes co-expressed with *BrNPF7.6* and *BrNPF8.9* (Figure 5C). Co-expression analysis and reference to *Arabidopsis* revealed that several pollen coats or tapetum development-related genes were co-expressed with *BrNPF2.6* and *BrNPF8.9*, including *BrSHT* (homolog of *Arabidopsis Spermidine Hydroxycinnamoyl Transferase*), *BrA7* (homolog of *Arabidopsis Thaliana Anther 7*), and *BrPTEN1* (homolog of *Arabidopsis Phosphatase And Tensin Homolog Deleted On Chromosome Ten 1*) (Figure 5D) [45,46,47]. In addition, *BrPRK2* (homolog of *Arabidopsis Pollen Receptor such as Kinase 2*), *BrARO1* (homolog of *Arabidopsis Armadillo Repeat Only 1*), *BrRABA4D* (homolog of *Arabidopsis Rab Gtpase Homolog A4d, Atraba4D*), and other important genes for pollen tube growth were represented in the genes co-expressed with *BrNPF7.6* and *BrNPF8.9* (Figure 5E) [48,49,50].

### 2.3. Expression of BrNPFs during B. rapa Growth during Vernalization and P. brassicae

**Figure 6:**
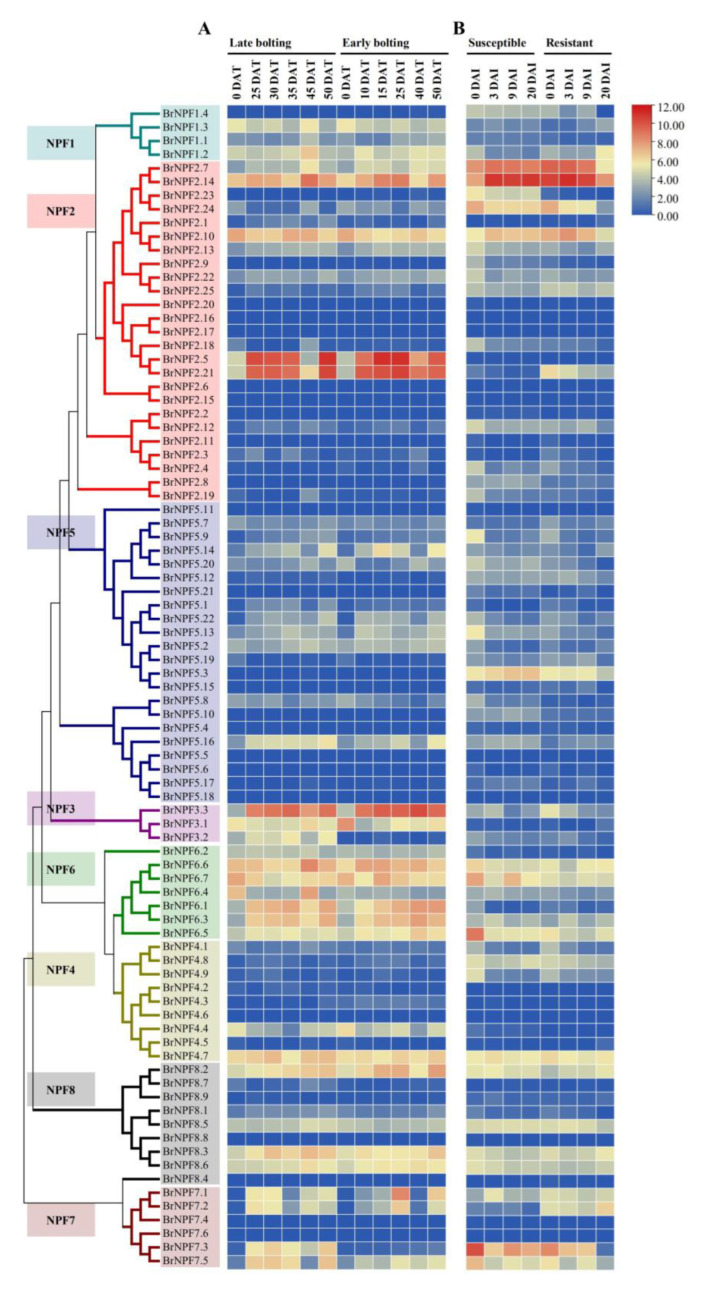
Expression patterns of *BrNPFs* responsive to vernalization (**A**) and *P. brassicae* infection stress (**B**). DAT, day after treatment. DAI, the day after inoculation.

*NPFs* have been previously suggested to be involved in plant responses during vernalization [21]. In order to identify *BrNPFs* that might be responsive to vernalization in *B. rapa*, RNA-Seq data for *BrNPFs* during vernalization were re-calculated, as previously described [51]. Briefly, *BrNPF2.5*, *BrNPF2.21*, *BrNPF3.3*, *BrNPF6.1*, *BrNPF6.3*, *BrNPF7.1*, and *BrNPF7.2* were up-regulated during vernalization in both genotypes, while *BrNPF7.3* and *BrNPF7.5* were only up-regulated in the late bolting type (Table S7 and Figure 6A). *BrNPF2.5* and *BrNFP2.21* are homologs of *AtNPF2.13* that are responsible for source-to-sink remobilization of nitrate [15], indicating that they might play important roles during nitrate remobilization during vernalization. *BrNPF3.3* is a homolog of *AtNPF3.1* that enables the transport of GA [28], indicating that *BrNPF3.3* might play important role in GA transport during the developmental phase transition.

The development of clubroot disease can be influenced by nitrogen fertilization [52], and NPFs are one of the main transporters of nitrogen, indicating that *NPFs* might be involved in a response mechanism to *P. brassicae* infection. RNA-seq data suggested that many NPF members were expressed after inoculation with *P. brassicae* (Table S7 and Figure 6B). Briefly, *BrNPF2.23* was only responsive to *P. brassicae* in the susceptible line, while *BrNPF5.3* was induced in the susceptible line after infection by *P. brassicae* (Figure 6B). *BrNPF2.21* and *BrNPF7.4* expressions were both up-regulated in the resistant line compared to the susceptible line (Figure 6B). *BrNPF2.24* exhibited down regulation in both the susceptible and resistant lines. Thus, *NPF* genes may participate in clubroot disease responses via the transport of their specific substrates.

### 2.4. Expression of BrNPF Responses to Low Nitrate Conditions

**Figure 7:**
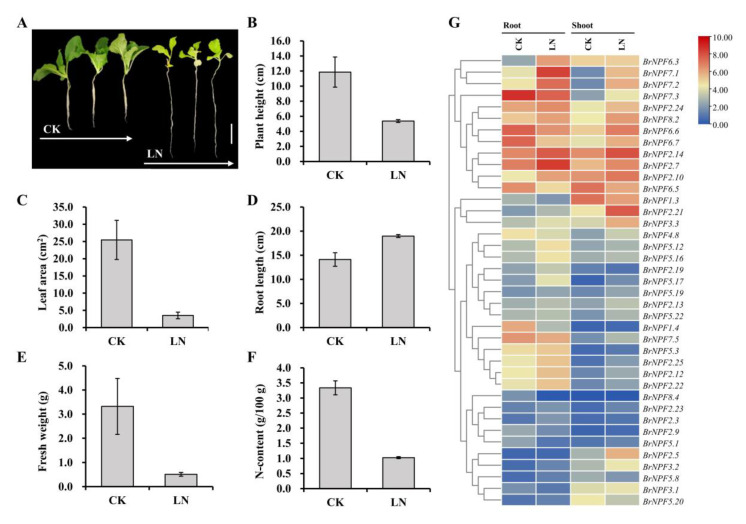
Phenotypic characterization and transcriptome profiling of *BrNPFs* under low nitrate availability stress. (**A**), *Brassica rapa* phenotype after low nitrate availability treatment. (**B**–**F**), Comparisons of plant height, leaf area, root length, fresh weight, and total nitrate concentrations between normal treatment and low nitrate-treated *B. rapa* seedlings. (**G**), Heatmap visualization of *BrNFPs* differential expression due to low nitrate stress.

Most *NPF* genes in *Arabidopsis* are related to nitrate transport, indicating that nitrate is the primary substrate of NPF proteins [53]. To assess the potential functions of *BrNPFs* in nitrate uptake and use, *B. rapa* seedlings (accession Chiifu-401-42) were hydroponically cultured in Hoagland’s nutrient solution [21] and treated with low nitrate conditions. After treatment, plant heights, leaf areas, fresh weights, and nitrogen contents were significantly lower, while root lengths increased compared to normal growth conditions (Figure 7A–F). The root and shoot components of seedlings were then separately sampled for RNA-seq analysis. Considering the criteria of TPM ≥ 1 and fold change values > 2.0, a total of 39 *BrNPFs* were identified as responsive to low nitrate conditions either in the shoots or roots (Table S8 and Figure 7G). Among these, ten *BrNPFs* (*BrNPF2.12*, *BrNPF2.14*, *BrNPF2.21*, *BrNPF2.22*, *BrNPF2.25*, *BrNPF2.7*, *BrNPF3.2*, *BrNPF5.17*, *BrNPF7.1*, and *BrNPF7.2*) were up-regulated in both shoots and roots under low nitrate conditions, while *BrNPF1.3* and *BrNPF6.5* were down-regulated in both shoots and roots. Twelve *BrNFPs* were specifically responsive to low nitrate conditions in shoots, of which eleven genes (*BrNPF2.13*, *BrNPF2.23*, *BrNPF2.24*, *BrNPF2.5*, *BrNPF3.3*, *BrNPF4.8*, *BrNPF5.19*, *BrNPF5.22*, *BrNPF5.3*, *BrNPF7.5*, and *BrNPF8.2*) were up-regulated, indicating their potential positive function in nitrate homeostasis (Table S8 and Figure 7G). Seven genes (*BrNPF2.10*, *BrNPF2.19*, *BrNPF2.3*, *BrNPF5.12*, *BrNPF5.16*, *BrNPF5.8*, and *BrNPF6.3*) were only up-regulated in roots indicating that they may play positive roles in nitrate absorption. In addition, five *BrNFPs* (*BrNPF1.4*, *BrNPF2.9*, *BrNPF3.1*, *BrNPF5.1*, and *BrNPF8.4*) were down-regulated in roots, suggesting they may play negative roles in nitrate absorption (Table S8 and Figure 7G). Three *BrNPFs* (*BrNPF6.6*, *BrNPF6.7*, and *BrNPF7.3*) were up-regulated in shoots, but down-regulated in roots, indicating that they may exhibit contrasting roles in roots and shoots during low nitrate conditions.

## 3. Discussion

### 3.1. Identification and Analysis of BrNPFs

*NFPs* are one of the largest groups of transporter family genes in plants. Genome-wide identification of *NFP* family genes has been conducted in many plants based on sequence and motif conservation, including in *Arabidopsis* [15], rice [54], apple [17], sugarcane [18], *Brassica napus* [19,20,21], hexaploid wheat (*Triticum aestivum* L.) [22], spinach (*Spinacia oleracea* L.) [23] and tea plants (*Camellia sinensis*) [24]. In this study, 85 *NFPs* were identified in *B. rapa*, in addition to 110 members in *B. oleracea* and 97 in *B. nigra* (Table S1). Ninety-five *NPFs* were previously identified in *B. rapa* (“Chiifu-401”, version 1.5) [1]. The differences in identification could be explained by the use of a higher quality genome version (“Chiifu-401”, version 3.0) [55], leading to greater accuracy in *BrNPF* identification. Similar results were also observed for *B. napus*, in which 169, 193, and 199 *NPFs* were identified based on different versions of *B. napus* genomic data [19,20,21]. *Brassica* species have undergone an extra genomic duplication event compared to *Arabidopsis* [56]. Thus, one *Arabidopsis* gene should theoretically have one to three orthologs in *Brassica* genomes. However, the expansion of *NPFs* in *B. rapa*, *B. oleracea*, and *B. nigra* represent 1.6-, 2.0, and 1.8-fold increases relative to the *Arabidopsis* genome, respectively. Thus, duplicated genes may have been lost during *Brassica* evolution. Consistently, *AtNPF2.1*, *AtNPF2.2*, and *AtNPF2.5* did not have any orthologs in the three species of *Brassica* analyzed here, while six, six, and four orthologs of *AtNPF5.2* were identified in the *B. rapa, B. oleracea*, and *B. nigra* genomes, respectively (Figure 1 and Table S1).

Segmental duplication and tandem duplication are two major mechanisms of gene family duplication in plants [57]. In this study, 22 tandem duplicated genes (25.9%) were identified in the *B. rapa* genome, while no segmental duplication events were identified (Figure S1). Thus, tandem duplication may be a primary driving force in the expansion of *BrNPFs*. In contrast, six segmental duplication events were observed within the genome of *B. oleracea,* which may explain the greater number of *NPFs* in the *B. oleracea* genome compared to the *B. rapa* and *B. nigra* genomes (Figure 3).

### 3.2. BrNPF Functions

*NPF* genes represent one of the largest transporter gene families in plants and participate in the transport of diverse substrates across membranes over short or long distances [11,19]. *Cis*-elements play key roles in controlling gene expression during plant development and are responsive to stress [58]. Numerous *cis*-elements have been previously observed in the promoter regions of *NPFs* from *B. napus*, tea plant, apple, and spinach plants, in addition to others [14,19,20,23,52]. In the present study, many phytohormone-responsive, environmental-responsive, plant growth and development-related, and transcriptional factor binding *cis*-elements were identified in the promoters of the *BrNPFs* (Table S4). The existence of abundant and diverse *cis*-elements in *NPF* gene promoters could be related to their multiple functions during plant development and responses to environmental stresses. 

Gene expression patterns can provide clues for predicting gene functions. Consequently, expression profiles for *BrNPFs* from 59 diverse tissues were analyzed, in addition to expressional profiles responsive to vernalization and *P. brassicae* infection between contrasting genotypes and under low nitrate availability stress (Figure 4, Figure 6 and Figure 7). Some *BrNPFs* exhibited tissue-specific expression, while others exhibited differential expression due to vernalization, *P. brassicae* infection, and low nitrate availability. For example, the homolog of *AtNPF3.1*, *BrNPF3.3,* exhibited induction by vernalization (Figure 6A). Nitrate was previously reported to delay flowering time via the GA signaling pathway [59]. Further, *AtNPF3.1* in *Arabidopsis* has been reported to be involved in GA transport [60], with GA increasing during *B. rapa* vernalization [61]. The up-regulation of *BrNPF3.3* due to vernalization suggests that it might contribute to GA presence during vernalization. Further, nitrogen fertilization has been shown to affect the susceptibility of *B. napus* [52]. Homologs of *AtNPF2.13* and *BrNPF2.21* exhibited up-regulation in the resistant genotype (Figure 6B). *AtNPF2.13* is involved in the remobilization of nitrate from sources to sinks [62], indicating that *BrNPF2.21* might respond to clubroot disease through the redistribution of nitrate. These results collectively provide new insights for the future functional prediction and characterizations of *BrNPFs*.

### 3.3. BrNPFs and Pollen Development

*AtNPF2.8* was previously suggested by co-expression analysis to be involved in the transport of flavonol-3-O-sophoroside from tapetum cells to pollen walls in *Arabidopsis* [13]. Two orthologs of *AtNPF2.8* were identified in *B. rapa*, including *BrNFP2.6* and *BrNPF2.15* (Figure 1 and Table S1). Both orthologs exhibited expression only in fertile floral buds of MS lines (Figure 5B). Further, *BrNPF2.6* was highly expressed in floral buds containing pollen grains from tetrads prior to the mature stage (from the F2 to F3 stages). In addition, *BrNPF2.15* only exhibited expression in F3-stage floral buds that contain pollen grains after the tetrad to before the mature stages (Figure 5B). Additionally, gene co-expression profiles and associated GO enrichment biological processes differed between *BrNFP2.6* and *BrNPF2.15*. For example, pollen development was represented by genes co-expressed with *BrNFP2.6*, while pollen tube development was suggested by genes co-expressed with *BrNPF2.15* (Figure 5C,D). Thus, the function of *AtNPF2.8* might have expanded or diversified over *Brassica* evolution. Flavonol diglucosides are essential for maintaining pollen fertility and increasing pollen tolerance to environmental stresses [63]. Thus, expanded *AtNPF2.8* genes suggest that the regulatory network related to flavonol diglucoside metabolism during pollen development might be more complex in *B. rapa* compared to *Arabidopsis*.

*BrNFP7.6* and *BrNFP8.9* were also specifically expressed in fertile floral buds (Figure 4 and Figure 5B) and were the only identified orthologs of *AtNPF7.1* and *AtNPF8.2*, respectively (Figure 1). *AtNPF8.2* is also referred to as *AtPRT5* and facilitates peptide transport into germinating pollen and possibly into maturing pollen, ovules, and seeds [36]. Pollen development and pollen tube development were suggested by genes co-expressed with *BrNFP8.9* (Figure 5C–E). Based on the phylogenetic and Semi-quantitative RT-PCR analysis results, the functions of *AtNPF8.2* and *BrNFP8.9* may be highly conserved during pollen development and pollen tube growth. The processes of pollination, pollen tube growth, lipid oxidation, transport, and response to nutrient levels were also represented by genes co-expressed with *BrNPF7.6*, indicating that they might also play important roles in pollen development. Taken together, the fertile floral buds exhibited genes that were specifically expressed in these tissues, and these may provide new breeding targets for creating sterile male lines in *B. rapa*.

### 3.4. Responses of BrNPFs during Low Nitrate Stress

Nitrate was previously identified as the main substrate of *NPF* genes [22,53]. Here 45.9% of *BrNPFs* (39 of 85) exhibited differential expression due to low nitrate availability stress (Figure 7G). Among these, ten were induced in both shoots and roots, eleven were specifically induced in shoots, and seven were only up-regulated in roots, indicating that they might play positive roles in nitrate absorption, uptake, homeostasis, and redistribution under low nitrate availability conditions (Table S8 and Figure 7G). *AtNPF2.3* in *Arabidopsis* functions as a root stele transporter and contributes to nitrate translocation to shoots during salt stress [12]. In this study, a homolog of *AtNPF2.3*, *BrNPF2.3*, exhibited specific induction in roots, indicating that it might serve a similar function (Figure 1 and Figure 7G). *AtNPF6.2* plays key role in regulating leaf nitrate homeostasis [32]. The expression of its homolog, *BrNPF6.5*, was repressed in both shoots and roots, indicating that low nitrate availability may lead to decreased NPF transporter activity (Figure 1 and Figure 7G). *AtNPF2.13* is reportedly involved in the transport of nitrate and GA [15]. A homolog of *AtNPF2.13*, *BrNPF2.21*, was up-regulated in both shoots and roots, indicating that *BrNPF2.21* might function in response to low nitrate availability by coupling to hormone signaling. *AtNPF6.3* can repress lateral root growth under low nitrate availability by promoting basipetal auxin transport out of roots [3]. In this study, a homolog of *AtNPF6.3*, *BrNPF6.6*, was down-regulated in roots but up-regulated in shoots (Figure 1 and Figure 7G). Taken together, the expression of *BrNPFs* under low nitrate availability conditions suggests that there is a crosstalk between low nitrate stress responses and phytohormone signaling pathways, consistent with results from previous studies [59].

## 4. Materials and Methods

### 4.1. Plant Growth and Low Nitrate Treatments

Uniform *B. rapa* seeds (accession Chiifu-401-42) were germinated in Petri dishes at 23 ± 1 °C in the dark for two days, followed by hydroponic cultivation of germinated seeds in Hoagland’s nutrient solution for one week [64]. To establish low nitrate treatments, KNO_3_ and Ca(NO_3_)_2_ in Hoagland’s solutions were replaced with KCl and CaCl_2_, respectively. The final concentration of NO_3_^−^ in the treatments was 0.1 mM. During cultivation, growth conditions within growth chambers were set as previously described [65]. After treatments, the shoots and roots in the low nitrogen and control treatments were individually harvested and immediately frozen in liquid nitrogen, followed by storage at −80 °C.

In order to collect materials from male sterile (MS) lines, seeds of MS lines from our previous study were germinated in Petri dishes at 23 ± 1 °C in the dark [40]. Vernalization was then induced with germinated seeds at 4 °C in the dark for 30 days. After vernalization, seeds were sown into pots (15 × 15 × 18 cm) containing potting soil and transferred to a greenhouse, followed by growth at 23 ± 1 °C with a light intensity of 6000–7000 Lux under a long day photoperiod (light/dark, 16 h/8 h). After flowering, floral buds were collected from MS line plants using three biological replicates and with previously reported criteria [40]. Root and shoot tissues were collected from three-week-old seedlings without vernalization. Stem and leaf tissues were sampled from plants one week after bolting. The siliques were collected two weeks after pollination. After sampling, tissues were immediately frozen in liquid nitrogen and stored at −80 °C until further analysis.

### 4.2. Identification of BrNPFs in Three Prototypical Diploid Species of Brassica

To identify NPF proteins from three prototypical diploid species of *Brassica*, all putative protein sequences encoded by the *B. rapa* (“Chiifu-401”, version 3.0) [55], *B. oleracea* (“JZS,” version 2.0) [66] and *B. nigra* (“Ni100”, version 2.0) [67] genomes were downloaded from the Brassicaceae Database (BRAD; www.brassicadb.cn accessed on 20 April 2022). The previously identified 53 AtNPFs were used as queries to search against *Brassica* protein sequences (using an *E*-value < 10^−5^ and identity > 20%). Search hits without a proton-dependent transport 2 (PTR2) domain (PF00854) were excluded based on HMM analysis with an *E*-value cut-off of 10^−5^. In order to identify all potential NPFs among the three *Brassica*, the search baits were used as BLAST queries for searching against the Phytozome 13 and NCBI databases with an *E*-value < 10^−5^ and identity > 20%. No additional predicted NPFs were identified at this stage. All NPFs were identified according to previously reported rules [1].

### 4.3. Phylogenetic and Bioinformatic Analysis of BrNPFs

Phylogenetic analysis was conducted with the NPF protein sequences from *Arabidopsis*, *B. oleracea*, *B. nigra*, and *B. rapa* after alignment with the MUSCLE program, implemented in MEGA6 with default parameters [68]. An unrooted phylogenetic reconstruction was then constructed using MEGA6 with neighbor-joining methods and analysis parameters, including pairwise taxa deletion, 1000 bootstrap replicates, and the use of the Jones Taylor Thornton (JTT) amino acid substitution model [69]. The chromosomal positions of each NPF from the three genomes were identified among those from the BRAD (www.brassicadb.cn accessed on 16 August 2022) and visualized with a custom Python script. The isoelectric point (PI) and molecular weights (MWs) of the NPFs were analyzed using the ProtParam tool (Expasy, the Swiss Bioinformatics Resource Portal, https://web.expasy.org/protparam/ accessed on 16 August 2022) [70]. Subcellular localization predictions of NPFs were conducted using the WoLF PSORT software package (https://wolfpsort.hgc.jp/ accessed on 16 August 2022) with default settings. Conserved motifs in the BrNPFs were identified using the MEME software program (Suite 5.1.1, http://meme-suite.org/ accessed on 16 August 2022) [71]. BrNPFs gene structures were drawn using the Gene Structure Display Server (GSDS; version 2.0, http://gsds.cbi.pku.edu.cn/ accessed on 16 August 2022) [72].

### 4.4. RNA Extraction, Leaf Area, and Nitrate Content

Total RNA was isolated from 100 mg of homogenized leaves using the RNAiso Plus Reagent (Takara Biomedical Technology Co., Ltd., Beijing, China) according to the manufacturer’s instructions. The outermost leaves of Chiifu seedlings were dissected for leaf area determination using a Yaxin-1241 leaf meter (Beijing Yaxinliyi, Beijing, China), following the manufacturer’s instructions. The nitrogen concentrations of oven-dried shoots were measured using the Kjeldahl method with a JK9830 Kjeldahl Auto Analyzer (ELITE-Lab Instrument Co., Ltd., Jinan, China) [73] and are expressed as concentrations of per hundred dry matter (g/100 g).

### 4.5. RNA-Sequencing and Assembly

RNA samples from low nitrate treatments were sent to Gene Denovo Biotechnology Co., Ltd. (Guangzhou, China) for RNA-Seq analysis. The RNA libraries were constructed and sequenced on the Illumina platform. Sequencing and analyses were conducted following standard protocols at Gene Denovo Biotechnology Co., Ltd. (Guangzhou, China). Filtered clean reads were then mapped to the reference genome (“Chiifu-401”, version 3.0) using the HISAT2 software program [74], and transcripts per million (TPM) values were calculated using DESeq [75]. Raw sequencing data were deposited in the China National Center for Bioinformation (CNCB) under the project ID PRJCA012597.

### 4.6. Expression of BrNPFs within RNA-Seq Data

To analyze the expression of *BrNPFs* among various tissues, publicly available RNA-seq data from 59 different organs or tissues of *B. rapa* were retrieved from the NCBI database via the Bioproject accessions PRJNA185152, PRJNA778186, PRJNA641876, and PRJNA473318 [76,77,78,79]. Data were included from tissues comprising callus, stems, stem leaves, opened flowers, siliques, different developmental stages of heading leaves, floral buds, pistils, embryos, and seed coats. Gene expression levels were re-calculated using the transcripts per million (TPM) metric. Heatmaps for expression profiles of *BrNPFs* were generated using the TBtools software program (version 1.0987663) [80].

The expression of *BrNPFs* after vernalization was recalculated for a previous study (BioProject PRJNA615255) using the TPM metric [51]. To achieve vernalization, two inbred *B. rapa* accessions, including a late bolting type (JWW) and an early bolting type (XBJ), were investigated. Prior to vernalization, both inbred lines were grown at 25 ± 2 °C for 32 days under natural light conditions. Both inbred lines were then transferred to a growth chamber at 4 °C with 150 µmol m^−2^s^−1^ light intensity under long daylight conditions (16/8 h, day/night) for vernalization, followed by a collection of the third fully expanded leaves from the center for subsequent analyses. JWW leaves were collected after 0, 25, 30, 35, 45, and 50 days following treatment. XBJ leaves were collected 0, 10, 15, 25, 40, and 50 days after treatment.

The TPM values of *BrNPF* genes were recalculated after infection with *Plasmodiophora brassicae,* as described in previous studies (Bio-Project PRJNA743585) [81]. In order to initiate *P. brassicae* infection, 20-day-old healthy plants of resistant (BrT24) and susceptible (Y510-9) *B. rapa* genotypes were inoculated with 20 mL of a *P. brassicae* (race 4) solution. For the control group, 20 mL of sterile water was used for inoculation. The root samples for each genotype were then collected at 0, 3, 9, and 20 d after inoculation, based on the four-time points of disease development [81].

### 4.7. Semi-Quantitative RT-PCR 

First-strand cDNA was synthesized using the PrimeScript™ RT reagent Kit (Takara Biomedical Technology Co., Ltd., Beijing, China) using 1 μg of total RNA. Synthesized cDNA was then diluted to 10 ng/μL for PCR amplification. Semi-quantitative RT-PCR assay reactions (20 µL) contained: 2 µL (20 ng) template cDNA, 1.0 µL (10 pmol) of forward primer, 1.0 µL (10 pmol) of the reverse primer, 10 µL 2 × Tag PCR StarMix (GenStar Biosolutions Co., Ltd., Beijing, China) and 6 µL distilled water. The thermocycling conditions were: denaturation at 94 °C for 5 min, followed by 94 °C for 30 s with 28 cycles, then 55 °C for 30 s, and 72 °C for 60 s. PCR primer sequences used for semi-quantitative RT-PCR are shown in Table S9. Semi-quantitative RT-PCR products were separated on 1.5% agarose gels and stained with ethidium bromide to evaluate amplification success.

## 5. Conclusions

Here, a total of 85, 110, and 97 NFP proteins were identified in the genomes of *B. rapa*, *B. oleracea*, and *B. nigra*, respectively. The gene structures, chromosomal locations, conserved motifs, *cis*-elements, evolutionary relationships, gene duplications, and expression patterns of the *BrNFPs* were systematically analyzed. These results provide new targets for future studies to elucidate the molecular mechanisms underlying *BrNPF* functions in pollen development, nitrate utilization, responses to vernalization, and *P. brassicae* infection response in *B. rapa*, especially for *BrNPF2.6*, *BrNPF2.15*, *BrNPF7.6*, and *BrNPF8.9* showing potential for generating sterile male lines based on gene editing methods in *B. rapa* and, possibly, other crops.

## Data Availability

The datasets generated or analyzed in the present study are available in the supplementary data or within the CNCB (China National Center for Bioinformation) under project ID PRJCA012597.

## Supplementary Materials

The following supporting information can be downloaded at: https://www.mdpi.com/article/10.3390/ijms24010754/s1.
