# Supplementary material for: Identification of NPF Family Genes in Brassica rapa Reveal Their Potential Functions in Pollen Development and Response to Low Nitrate Stress
Source: Int J Mol Sci. 2023 Jan 1;24(1):754. doi: 10.3390/ijms24010754 (PMC9821126; doi:10.3390/ijms24010754)
Supplement: Supplementary file 1 [file ijms-24-00754-s001.zip › Table S2.pdf]

Table S2. Conserved motifs identified in BrNPF proteins.

Motif No.	Motif consensus sequence	*E*-value	Motif Width	No. of *BrNPF* proteins	Motif annotation
1	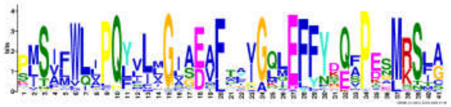	6.3e-1603	41	75	PTR2 domain (PF00854)
2	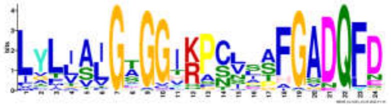	1.4e-873	24	72	PTR2 domain (PF00854)
3	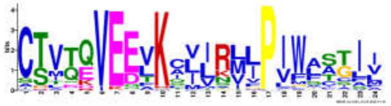	7.6e-816	24	71	PTR2 domain (PF00854)
4	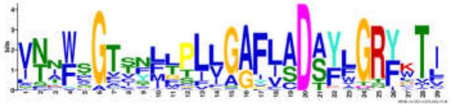	2.5e-1022	29	77	PTR2 domain (PF00854)
5	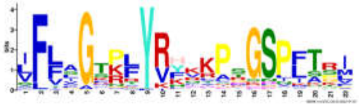	1.7e-578	22	**78**	PTR2 domain (PF00854)
6	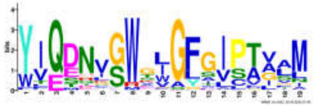	4.9e-572	19	74	PTR2 domain (PF00854)
7	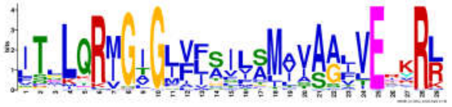	1.1e-918	29	**73**	—

8	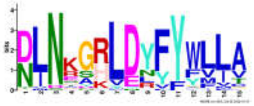	2.9e-530	15	71	—
9	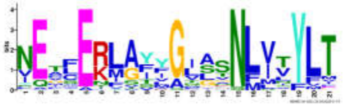	1.2e-517	21	65	—
10	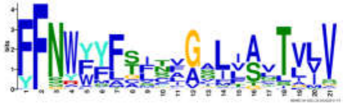	1.4e-555	21	72	PTR2 domain (PF00854)
