# Supplementary figures and images for: Identification of NPF Family Genes in Brassica rapa Reveal Their Potential Functions in Pollen Development and Response to Low Nitrate Stress
Source: Int J Mol Sci. 2023 Jan 1;24(1):754. doi: 10.3390/ijms24010754 (PMC9821126; doi:10.3390/ijms24010754)

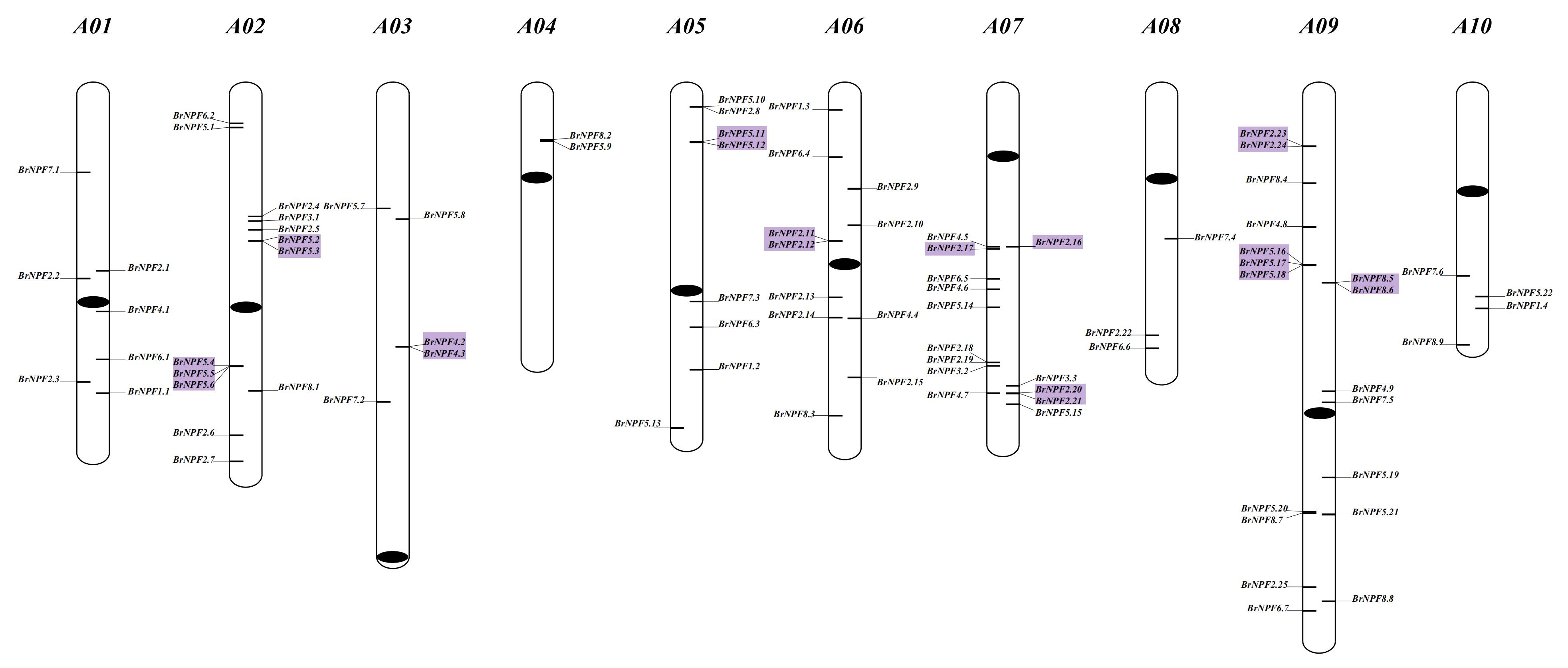

Supplement: Supplementary file 1 [file ijms-24-00754-s001.zip › Figure S1.jpg]
